# Exploring Cannabinoids with Enhanced Binding Affinity for Targeting the Expanded Endocannabinoid System: A Promising Therapeutic Strategy for Alzheimer’s Disease Treatment

**DOI:** 10.3390/ph17040530

**Published:** 2024-04-19

**Authors:** Gabriela Dumitrita Stanciu, Daniela-Carmen Ababei, Carmen Solcan, Cristina-Mariana Uritu, Vlad-Constantin Craciun, Cosmin-Vasilica Pricope, Andrei Szilagyi, Bogdan-Ionel Tamba

**Affiliations:** 1 Advanced Research and Development Center for Experimental Medicine “Prof. Ostin C. Mungiu”—CEMEX, Grigore T. Popa University of Medicine and Pharmacy, 16 Universitatii Street, 700115 Iasi, Romania; gabriela-dumitrita.s@umfiasi.ro (G.D.S.); andrei.szilagyi@umfiasi.ro (A.S.);; 2 Pharmacodynamics and Clinical Pharmacy Department, Grigore T. Popa University of Medicine and Pharmacy, 16 Universitatii Street, 700115 Iasi, Romania; 3 Faculty of Veterinary Medicine, “Ion Ionescu de la Brad” University of Life Sciences, 700490 Iasi, Romania; carmensolcan@yahoo.com; 4 Department of Computer Science, “Alexandru Ioan Cuza” University of Iasi, 700506 Iasi, Romania; craciun.vlad@yahoo.ie; 5 Department of Pharmacology, Clinical Pharmacology and Algesiology, Grigore T. Popa University of Medicine and Pharmacy, 16 Universitatii Street, 700115 Iasi, Romania

**Keywords:** Alzheimer’s disease, APP/PS1 transgenic mice, expanded endocannabinoid system, cannabinoid receptor ligands, chronic intermittent therapy, therapeutic tools

## Abstract

Despite decades of rigorous research and numerous clinical trials, Alzheimer’s disease (AD) stands as a notable healthcare challenge of this century, with effective therapeutic solutions remaining elusive. Recently, the endocannabinoid system (ECS) has emerged as an essential therapeutic target due to its regulatory role in different physiological processes, such as neuroprotection, modulation of inflammation, and synaptic plasticity. This aligns with previous research showing that cannabinoid receptor ligands have the potential to trigger the functional structure of neuronal and brain networks, potentially impacting memory processing. Therefore, our study aims to assess the effects of prolonged, intermittent exposure (over 90 days) to JWH-133 (0.2 mg/kg) and an EU-GMP certified *Cannabis sativa* L. (Cannabixir^®^ Medium Flos, 2.5 mg/kg) on recognition memory, as well as their influence on brain metabolism and modulation of the expanded endocannabinoid system in APP/PS1 mice. Chronic therapy with cannabinoid receptor ligands resulted in reduced anxiety-like behavior and partially reversed the cognitive deficits. Additionally, a reduction was observed in both the number and size of Aβ plaque deposits, along with decreased cerebral glucose metabolism, as well as a decline in the expression of mTOR and CB2 receptors. Furthermore, the study revealed enlarged astrocytes and enhanced expression of M1 mAChR in mice subjected to cannabinoid treatment. Our findings highlight the pivotal involvement of the extended endocannabinoid system in cognitive decline and pathological aspects associated with AD, presenting essential preclinical evidence to support the continued exploration and assessment of cannabinoid receptor ligands for AD treatment.

## 1. Introduction

Alzheimer’s disease (AD), a neurodegenerative condition marked by gradual cognitive decline and neurodegeneration, remains a pressing healthcare challenge, especially as the risk of dementia increases with the aging of the global human population and life expectancy [1,2]. Even after more than a century since its discovery and with the amyloid hypothesis being one of the well-established features of the disease, compressive therapeutic options continue to be limited. As such, the available drug classes aimed at addressing the pathology and clinical profile of AD predominantly focus on improving memory by inhibiting the acetylcholinesterase (AChE) enzyme [3,4,5]. Additionally, the efficacy of anti-amyloid-β monoclonal antibodies (Aducanumab, Lecanemab, Crenezumab, Bapineuzumab, Solanezumab, and Gantenerumab) appears to provide only marginal clinical benefits [6,7]. However, AD is not solely attributed to a single factor like AChE inhibition; instead, it is a multifactorial condition. Factors like oxidative stress, neuroinflammation, neurotransmitter imbalance, and mitochondrial and synaptic dysfunction are also key contributors to cognitive decline [8,9]. Consequently, the most effective therapy for AD should be able to influence the disease via multiple pathways rather than targeting a single dysregulated mechanism.

Experimental and clinical data have revealed a key role of the endocannabinoid system (ECS) in a wide range of neural functions, including learning and memory, motor coordination, emotional processing, motivation, and regulation of GABAergic and cholinergic neurons [10]. The ECS consists of two main receptor subtypes: the cannabinoid receptor types 1 and 2 (CB1 and CB2), along with endocannabinoids (eCBs) and the enzymes involved in the production and degradation of eCBs [3]. Ongoing research has been directed towards investigating the ECS by using cannabinoid receptor ligands, such as tetrahydrocannabinol (THC, the primary psychoactive compound found in cannabis) or cannabidiol (CBD, a key component of cannabis), to discover the role that ECS might play in neurodegenerative disorders [11,12,13]. Under inflammatory conditions, CB2 receptors are particularly recognized as primary regulators of microglial activity, exhibiting an upregulation [14]. 

In addition, expanded ECS, known as the “*endocannabinoidome*”, has been recognized for its significance in elucidating the molecular mechanisms associated with various disorders and in the development of novel therapeutics. The endocannabinoidome includes, theoretically, a complex array of lipid mediators, metabolic enzymes, and receptor proteins. Furthermore, the endocannabinoidome includes various molecular targets, such as cannabinoid receptors (CB1 and CB2), orphan G protein-coupled receptors (GPCRs), transient receptor potential (TRP) channels, and peroxisome proliferator-activated receptors (PPARs) [15,16]. For example, the mechanistic target of rapamycin (mTOR) is a signaling protein that belongs to the endocannabinoidome, which has recently gained attention for its role in beta-amyloid (Aβ) and tau-induced neurodegeneration [17,18]. Besides, mTOR signaling has the capacity to modulate endocannabinoid synthesis and metabolism. Research indicates that mTOR activation can enhance the production of endocannabinoids and alter their levels in different tissues [3,19]. However, the precise interplay between endocannabinoid signaling and mTOR pathways in cognitive decline remains to be fully elucidated. The activation of M1 muscarinic acetylcholine receptors (M1 mAChRs), which play a central role in mediating the effects of acetylcholine, has been observed to impact endocannabinoid signaling. This interaction highlights the integration of M1 mAChRs within the broader framework of the endocannabinoidome, where lipid-derived signaling molecules and their receptors interact with various neurotransmitter systems. Conversely, endocannabinoids have been shown to exert regulatory influence on cholinergic signaling pathways, highlighting the bidirectional communication within the endocannabinoidome [20,21].

Since the early stages of AD are associated with a gradual and sustained decline in neuronal function, exploring novel perspectives of neuroinflammation and neuroprogression by the expanded ECS may offer insights into the underlying molecular processes of AD. In light of all this data, our research aimed to evaluate the effects of a chronic, intermittent exposure, to two cannabinoid receptor ligands (JWH-133 and Cannabixir^®^ Medium Flos—an EU GMP-certified batch of a dried inflorescence of *Cannabis sativa* L. with 15.6% THC and <1% CBD) on recognition memory, as well as their influence on brain metabolism and modulation of the expanded endocannabinoid system in APP/PS1 mice.

The selection of JWH-133 and Cannabixir^®^ Medium Flos—an EU GMP-certified batch of a dried inflorescence of *Cannabis sativa* L.—as the drug candidates for our study was guided by scientific evaluation and the need to fill knowledge gaps. The Cannabixir^®^ Medium Flos was chosen due to its promising pharmacokinetic profile, which was thoroughly investigated in toxicity studies conducted by our research team [22]. Also, it has been subject to rigorous quality control procedures in accordance with EU-GMP standards. This guarantees its consistency and reliability for reproducible medical and therapeutic applications. In contrast, JWH-133, while promising in short-term studies [23,24], lacked long-term data, prompting our investigation to address this gap. Additionally, our study aims to provide insights into mitigating ‘on-target’ side effects frequently associated with chronic cannabis use. This approach aligns with our overarching objective of advancing the field of cannabinoid therapeutics and enhancing patient care. 

## 2. Results

### 2.1. General Health Parameters

**Figure 1:**
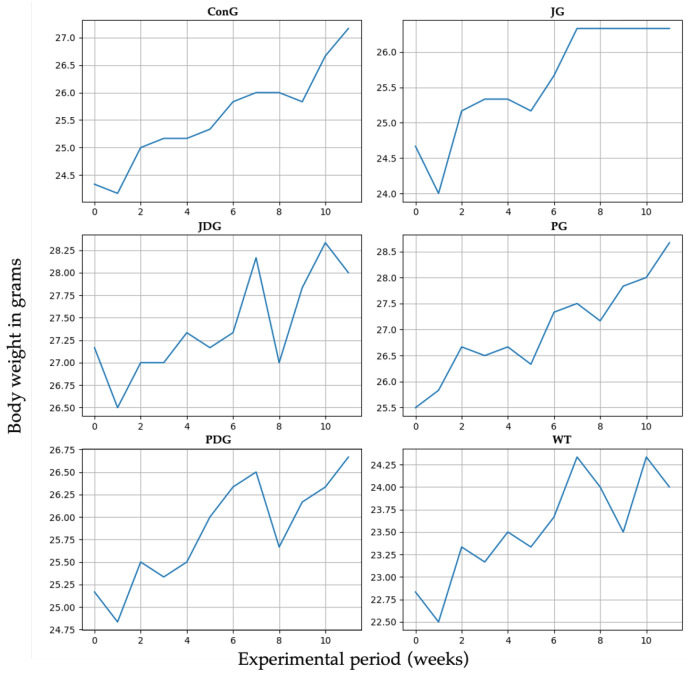
Effect of the CB2 receptor agonist JWH-133 and Cannabixir^®^ Medium Flos on weight gain. Throughout the 12-week treatment period, the body weight of all animals in each group was measured weekly. The groups were designated as follows: ConG: control group; JG: JWH-133 group; JDG: JWH-133 + donepezil group; PG: Cannabixir^®^ Medium Flos group; PDG: Cannabixir^®^ Medium Flos + donepezil group; WT: vehicle group. The substances were administered via gavage at a volume of 0.5 mL/100 g of body weight as follows: JWH-133 at a dose of 0.2 mg/kg, Cannabixir^®^ Medium Flos at a dose of 2.5 mg/kg, donepezil at a dosage of 0.65 mg/kg, and a 0.1% aqueous suspension of CMC-Na used as a vehicle.

The impact of cannabinoid receptor ligand therapy was evaluated in mice fed a standard diet. 90-day therapy with CB2 receptor agonist JWH-133 (0.2 mg/kg) or Cannabixir^®^ Medium Flos (2.5 mg/kg) did not significantly affect weight gain at the doses studied. The trendline for the ConG group is continuously growing from 24.5 to 27 g, which may be taken as a normal weight evolution. Quite remarkable is that all the other groups display spikes and falls in weight dynamics without statistical significance (*p* > 0.05). The JG group weight gain shows a very early ascending trendline, reaching 27 g from the 6–7 week and plateauing for the remaining time period of the experiment. The JDG and PDG groups exhibit an acute fall in the 8th week, followed by a quick recovery in the next few weeks. The WT group dynamics are also marked by numerous variations as compared to the ConG (Figure 1).

### 2.2. The Activation of the Expanded Endocannabinoid System Using Cannabinoid Receptor Ligands Reduced Anxiety-like Behavior and Partially Reversed the Cognitive Impairment in AβPP/PS1 Mice

**Figure 2:**
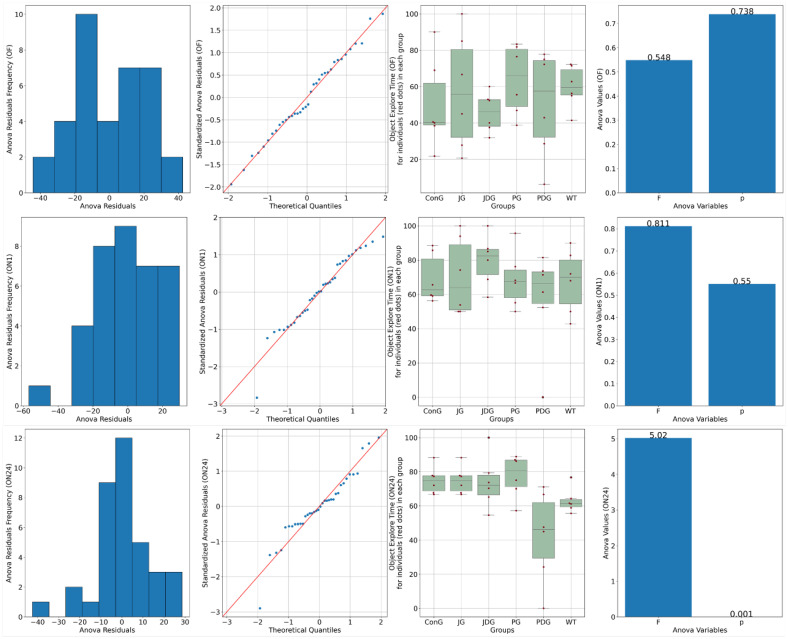
A novel object recognition (NOR) test was performed to detect the cognitive impairment of APP/PS1 mice. Data are represented as % of exploration time. On the training session (OF), the % of time spent exploring each of the familiar objects did not differ between control and cannabinoid receptor ligand-treated animals (*p* > 0.05). On the test day, 24 h following the training period, JG, JDG, and PG mice explored the novel object significantly more than the familiar object (*p* < 0.05), which indicated that cannabinoid receptor ligand therapy can improve NOR memory deficit in APP/PS1 mice. OF: training session using two identical objects (familiar objects); ON1: test session—evaluation of short-term memory, after an interval of 60 min; ON24: test session—evaluation of long-term memory, 24 h following the training period; ConG: control group; JG: JWH-133 group; JDG: JWH-133 + donepezil group; PG: Cannabixir^®^ Medium Flos group; PDG: Cannabixir^®^ Medium Flos + donepezil group; WT: vehicle group.

Chronic, intermittent administration of the two cannabinoid receptor ligands showed partial reversal of the cognitive deficits assessed in the NOR test. In the training phase, both control and treated animals spent equal amounts of time exploring the objects (*p* > 0.05). On the test day, 1 h following the training period, ConG and WT have values of preference for the novel object close to 70%, which are doubled by the JG and PG groups with 68.3 and 70%, respectively. The PDG shows a deterioration of the memory in this task with a reduction of the exploration time of the novel object down to 56%, but JDG mice have an increased preference for the novel object compared to the control groups. On the test day, 24 h following the training period, JG, JDG, and PG animals explored the novel object significantly more than the familiar object (*p* < 0.05), which indicated that cannabinoid receptor ligand therapy may improve NOR memory deficits. However, the PDG group showed a clear memory impairment compared to the ConG group (*p* = 0.01), and surprisingly, the WT group showed some memory reduction compared to the ConG group (*p* = 0.02), which is difficult to explain (Figure 2).

**Figure 3:**
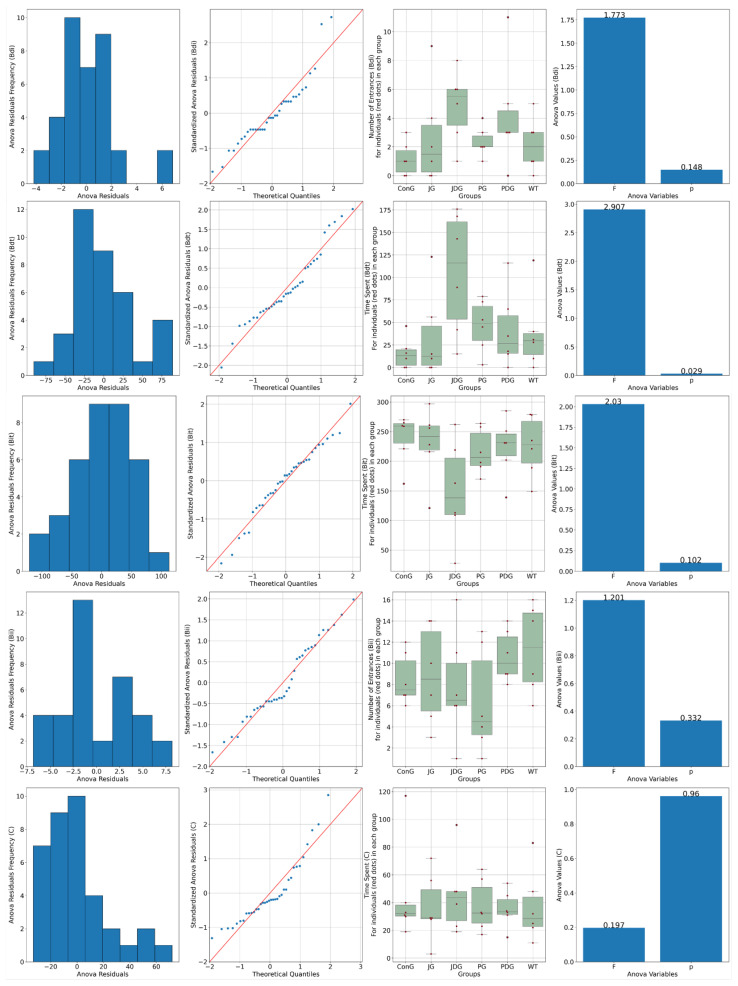
Effects of prolonged, intermittent cannabinoid receptor ligands on anxiety behavior in the elevated plus maze (EPM). Bdi-ConG had the lowest average Bdi of all the groups, with the JG group having a similarly low number of entrances. WT has slightly increased values compared to the ConG group. Two groups, JDG and PGD, display an increased Bdi compared to the ConG group. The WT group spends longer per average period of time in the open arms of the EPM compared to the ConG group. The PG group also shows disinhibited behavior compared to the ConG and WT groups. Bit: The ConG group spends up to 5/6 of the total time in the closed arms of the maze, the highest of all the groups, indicative of increased anxiety. The JG group also has a similar dynamic in this behavior to the ConG. The WT group has less inhibited behavior than the ConG, with 4/5 of the total time spent in the protected closed areas of the apparatus. There are only two groups, the PG and JDG groups, that spent shorter periods of time in the closed arms, exploring the exposed areas of the maze. For indicators Bdi, Bii, and Bit, there was not enough evidence to support a statistically significant difference among the groups (*p* > 0.05). Bdi: the time spent in the closed arms; Bdt: the time spent in the open arms; Bii: the entry frequency in the closed arms; Bdt: the entry frequency in the open arms; C: the time spent in the center of the maze; ConG: control group; JG: JWH-133 group; JDG: JWH-133 + donepezil group; PG: Cannabixir^®^ Medium Flos; PDG: Cannabixir^®^ Medium Flos + donepezil group; WT: vehicle group.

Anxiety-like behavior was measured after the memory test using the elevated plus maze (EPM). We found that the main indicator of anxiety, the time spent in the open arms (Bdt), varied between groups, and the ConG had the lowest values, reflecting either increased anxiety or inhibited behavior (Figure 3).

The JG group had similar values of Bdt to the ConG group. The WT group spends longer per average period of time in the open arms of the EPM compared to the ConG group. The least anxiety displaying features are visible in the JDG group, with similarly high values as in the open arm entry (Bdi) assessment (*p* < 0.05). The PG group also shows disinhibited behavior compared to the ConG and WT groups (*p* < 0.05)—Figure 3.

### 2.3. Prolonged Therapy with Selective CB2 Agonist JWH-133 or Cannabixir^®^ Medium Flos Reduces Cerebral Glucose Metabolism in APP/PS1 Mice

**Figure 4:**
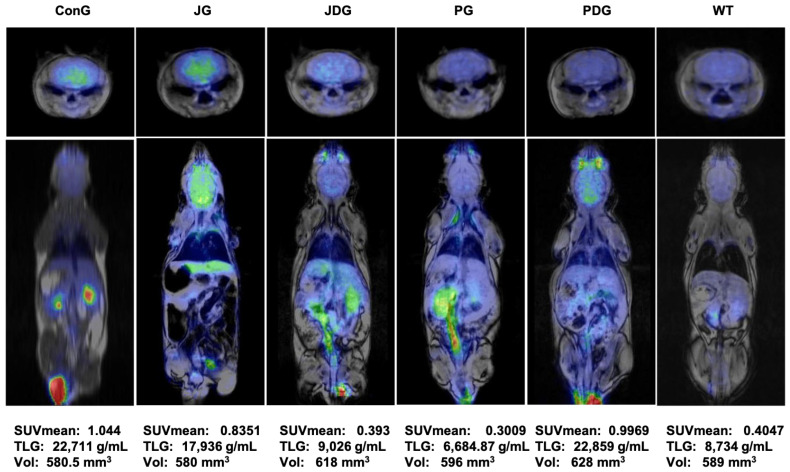
PET-MRI imaging data of a representative animal from each group in the axial (the upper line) and coronal (middle line) planes. The average values of SUVmean, TLG, and VOIs for each group are presented on the bottom line. PET-MRI: Positron Emission Tomography—Magnetic Resonance Imaging; SUV: standardized uptake values; TLG: Total Lesion Glycolysis, a quantitative measure that combines both the metabolic activity and the volume of a lesion identified on a PET scan; VOIs: volumes of interest.

To assess the impact of treatment on brain metabolism in vivo, all mice underwent ^18^F-fluorodeoxyglucose positron emission tomography (^18^F-FDG-PET) scans. Consistent with findings from prior studies, this research also reveals that in APP/PS1 mice, the presence of Aβ leads to a notably higher ^18^F-FDG uptake, as indicated by the values of SUVmean (the average standardized uptake value) and TLG (total lesion glycolysis) parameters. Figure 4 illustrates that in healthy mice (WT group), the average SUVmean was 0.4047, whereas in the ConG group, the average SUVmean was 1.044. All treated groups exhibited an improvement in health status, as evidenced by the monitored PET parameters.

Moreover, the ^18^F-FDG uptake in PG mice can be explained by a potential reduction of neuroinflammation associated with this model that may be successfully detectable with this radiotracer. According to the outcomes obtained in the present study, the groups JDG and PG responded most favorably to the medication administered to control AD.

### 2.4. The Effect of Pharmacological Modulation of the ECS on AD-Related Aβ Burden, Neuroinflammation, and Glial Reactivity in APP/PS1 Mice

To date, extensive research has explored the effects of pharmacological modulation of the ECS, demonstrating that activation of specific CB1 and CB2 receptors decreases the accumulation and deposition of Aβ plaques, reduces neuroinflammation, and improves cognitive decline in AD preclinical models [11,25,26,27,28,29]. However, due to the ambiguity regarding the precise role of cannabinoid receptors, our research investigated the effects of chronic, intermittent use of two distinct cannabinoid receptor ligands (CB2 receptor agonist JWH-13 and Cannabixir^®^ Medium Flos) on various markers closely associated with AD and the expanded endocannabinoid system, including the accumulation of Aβ plaque deposits, neuroinflammation, and glial reactivity. The quantitative IHC analysis of the biomarkers is shown in Table 1.

**Figure 5:**
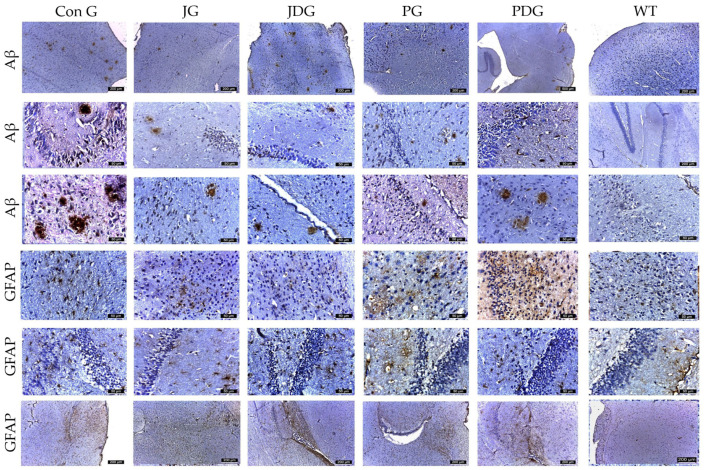
Representative immunohistochemical images of the Aβ plaques and astrocytes obtained from the control and treatment groups in both the cortex and hippocampus regions. The Aβ plaques that were identified appeared to be small, with an average size ranging from 30 to 50 µm^2^, except for those in the ConG group, which measured between 50 and 150 µm^2^. Across the different experimental groups, the average number of plaques followed a descending order as: ConG > JG > JDG > PDG > PG. GFAP-positive cells were observed in descending order as follows: PG, PDG, JG, JDG, ConG, and WT. Astrocyte numbers were assessed via GFAP staining. Notably, enlarged astrocytes were detected in the cerebral cortex, Ammon’s horn, and parahippocampal regions in the PG, PDG, JG, and JDG groups. In contrast, astrocytes in the ConG group were predominantly of medium and small sizes. Aβ: beta-amyloid; GFAP: glial fibrillary acidic protein; ConG: control group; JG: JWH-133 group; JDG: JWH-133 + donepezil group; PG: Cannabixir^®^ Medium Flos group; PDG: EU-GMP certified Cannabixir^®^ Medium Flos + donepezil group; WT: vehicle group.

In our study, cannabinoid therapy tended to decrease both the number and the size of the Aβ plaque deposits in APP/PS1 mice. The Aβ plaques that were detected appeared generally smaller, typically ranging in size from 30 to 50 µm^2^, except for those found in the ConG group, which showed measurements spanning from 50 to 150 µm^2^. Regarding the distribution of plaques among the different experimental groups, the average plaque count decreased in the following order: ConG > JG > JDG > PDG > PG. (Figure 5). Therefore, a numerical score ranging from 0 to 3 was assigned to assess the expression of amyloid plaque pathology, as follows: 0 (absence of plaques, WT mice), 1 (1 to 5 plaques/mm^2^, PG group), 2 (6 to 20 plaques/mm^2^, PDG, JDG, and JG animals), or 3 (more than 20 plates/mm^2^, ConG mice). Simultaneously, neurons expressing Aβ were found to be evenly spread across both the cortex and the parahippocampal area. However, their prevalence was significantly greater in ConG mice when compared to the treatment groups.

**Figure 6:**
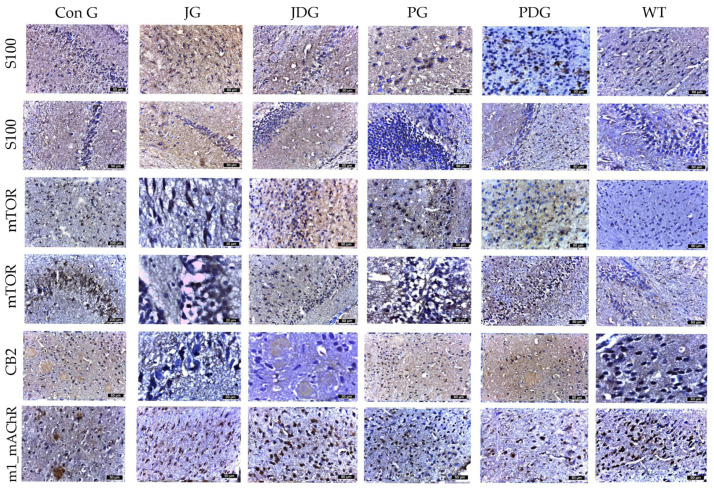
The effect of chronic, intermittent administration of the selective CB2 receptor agonist JWH-133 or Cannabixir^®^ Medium Flos, either alone or in conjunction with the donepezil, in the studied groups. Mouse brain tissue sections were sliced and immunoassayed for S100 to reveal any changes in microglia and astrocytes. The cells marked by S100 demonstrated numerical alignment with those identified by GFAP labeling. Additionally, they exhibited frequent, short, and robust extensions. The expression of mTOR and CB2 receptors was significantly increased in the frontal or parahippocampal cortex of ConG mice compared to the other experimental groups. Furthermore, a significant decrease in M1 mAChR expression was noted in ConG mice when compared to WT mice. S100: a nuclear transcription factor expressed by all astrocytes; mTOR: mammalian target of rapamycin; CB2: cannabinoid receptor type 2; M1-mAChR: muscarinic acetylcholine receptor M1; ConG: control group; JG: JWH-133 group; JDG: JWH-133 + donepezil group; PG: Cannabixir^®^ Medium Flos group; PDG: Cannabixir^®^ Medium Flos + donepezil group; WT: vehicle group.

Considering the dual function of glial cells in the brain—either mitigating Aβ-mediated neurotoxicity by improving its degradation and phagocytosis in normal conditions, as well as promoting neuroinflammation by triggering the release of inflammatory cytokines, thereby exacerbating neurodegeneration in AD patients [30,31], the effect of chronic cannabinoid therapy on astrogliosis was evaluated. Using GFAP and S100 to quantify reactive astrocytes, we observed a substantial increase in the number of astrocytes in the cerebral cortex, Ammon’s horn, and parahippocampal regions, ranging from 16–22 in the PG mice, 13–16 in the PDG animals, and 10–12 in the JG and JDG groups. In contrast, astrocytes in the ConG (6–10) group were predominantly medium- to small-sized (Figure 6). The cells marked by S100 demonstrated numerical alignment with those identified by GFAP labeling (Figure 5). Additionally, they exhibited frequent, short, and robust extensions. The expression of mTOR and CB2 receptors showed a significant increase in the subcortical, periventricular, and hippocampal areas of ConG mice compared to the other experimental groups. In the other groups (JG, JDG, and WT), the expression was reduced in the cortical area and was frequently absent in the hippocampus (Figure 6). Furthermore, a significant decrease in M1 mAChR expression was noted in ConG mice when compared to cannabinoid-treated and WT mice (Figure 6).

## 3. Discussion

The expanded endocannabinoid system is a relatively new focus of study, with ongoing exploration into its full therapeutic potential. As many symptoms of AD are related to cholinergic dysfunction [32,33], and coincide with changes in the endocannabinoid system [21], recent findings suggest that the potential crosstalk between these systems, acting as modulatory receptors, could provide an indirect method to regulate cholinergic dysfunction. This interaction is especially significant, particularly in the early stages of the disease, such as during the upregulation of CB2 receptor expression involved in the development of neuroinflammation [34]. However, targeting the ECS is not without its challenges, as CB1 and CB2 receptors serve as essential regulators of neuronal function, exerting varied effects on neuronal responses [35].

Our findings demonstrate that pharmacological activation of the expanded ECS via the selective CB2 agonist JWH-133 or Cannabixir^®^ Medium Flos may also be beneficial in neurodegeneration. Chronic intermittent therapy with these compounds in the pre-symptomatic APP/PS1 transgenic mice partially reversed the long-term recognition memory impairment and decreased anxiety-like behavior. In line with these results, Cakir et al. suggest that daily administration of JWH-133 (0.2 mg/kg, ip) for two weeks in an okadaic acid-induced rat model of AD decreases the disease’s pathological hallmarks and restores spatial memory impairment and anxiety to control levels [36]. Additionally, Aso et al. [25] showed that daily administration of JWH-133 (0.2 mg/kg, ip) for 5 weeks in APP/PS1 mice mitigates memory deficits, improves learning, and reduces neuroinflammation. However, earlier research has generated inconsistent data, with some indicating antianxiety effects and others suggesting anxiolytic effects following treatment with JWH-133 (3 mg/kg, ip) or THC (2, 4, and 8 mg/kg) [37,38,39]. Partially, this difference could be attributed to variations in species, strain, or genotype sensitivity to JWH-133 and THC, coupled with dosage differences. It’s intriguing to note that prolonged use of synthetic cannabinoids in humans has been correlated with increased symptoms of depression and anxiety [40].

In contrast to the typical findings observed in AD patients, but similar to several previous preclinical studies [41,42,43,44], we also noted an increased FDG uptake in the brains of the APP/PS1 transgenic mice (ConG). Our FDG-PET results demonstrate that chronic therapy with the selective CB2 agonist JWH-133 + donepezil (JDG mice) and Cannabixir^®^ Medium Flos (PG animals) leads to FDG uptake values comparable to those observed in WT mice. Consistent with the observed FDG outcomes, we noted a more pronounced reduction in both the number and size of Aβ plaque deposits in the same groups of mice (PG and JDG). Our data are in complete agreement with recent research showing that activating CB2 receptors promotes Aβ plaque removal [25,26,45], while deficiency in CB2 receptors in APP/PS1 mice markedly increases plaque deposition [46].

In parallel, enlarged astrocytes were detected in the cerebral cortex, Ammon’s horn, and parahippocampal regions in the PG, PDG, JG, and JDG animals. Conversely, astrocytes in the ConG group were predominantly of medium and small sizes. In AD, astrocytes undergo various changes, both morphologically and functionally. These alterations are described in the literature using terms such as (astro)gliosis, astrocyte activation, reactive astrocytes, and astrocyte reactivity [47,48]. Reactive astrocytes are evident in the APP/PS1 mice when plaques start to form, coinciding with cognitive decline [49,50]. However, much remains to be understood about the functional implications of their reactive state and their contribution to AD pathogenesis. Further research is needed to identify specific treatment windows and develop interventions targeting astrocytes for potential therapeutic strategies in AD.

Enlarged astrocytes in AD have been observed to surround and engulf Aβ plaques, the characteristic pathological hallmark of the disease. By phagocytosing Aβ, these astrocytes may help in reducing the burden of toxic protein aggregates in the brain, thereby exerting a neuroprotective effect [51,52]. Moreover, enlarged astrocytes may exert a neuroprotective role by modulating neuroinflammation. They can limit the release of pro-inflammatory cytokines and promote the clearance of inflammatory mediators, contributing to a more favorable microenvironment for neuronal survival [53]. Activated astrocytes can also secrete various neurotrophic factors, such as brain-derived neurotrophic factor (BDNF) and glial cell line-derived neurotrophic factor (GDNF). These factors play crucial roles in promoting neuronal survival, growth, and synaptic plasticity, counteracting the neurodegenerative processes seen in AD [54]. Additionally, astrocytes play a crucial role in regulating extracellular glutamate levels. ECS activation can modulate glutamate uptake and release by astrocytes, thereby preventing excitotoxicity and neuronal damage [55]. Furthermore, cannabinoid receptor activation may induce astrocyte hypertrophy, leading to enhanced astrocytic coverage of synapses and improved synaptic stability, which could confer neuroprotection [56]. Promoting Aβ clearance, secreting neurotrophic factors, maintaining brain homeostasis, modulating neuroinflammation, inducing astrocyte hypertrophy, and larger astrocytes in animals with AD treated with cannabinoids may indeed exert a neuroprotective role [57].

Furthermore, these findings were associated with an increase in mTOR expression and a decrease in M1mAChR expression observed in the cortex and hippocampus of mice in the ConG group, compared to mice treated with cannabinoids and WT animals. This reflects an impairment in the cholinergic transmission system observed from the mild cognitive impairment phase through the moderate pathological stages of AD [58].

The use of AD-transgenic mice serves as a valuable tool for assessing AD-related changes. However, it is critical to recognize that these models do not completely replicate all aspects of the human disease, thus constituting an inherent limitation in research. This limitation is acknowledged in the current study, which utilized one of the most prevalent animal models in AD research: the APP/PS1 mice. While APP/PS1 mice develop Aβ plaques and neurofibrillary tangles, they may not exhibit other pathological features seen in human AD, such as cerebral amyloid angiopathy, widespread neuroinflammation, or significant neuronal loss [59]. It is thus relevant to conduct more studies to define clinically significant doses and the therapeutic windows for cannabinoid receptor ligands, such as JWH-133 or Cannabixir^®^ Medium Flos. Following the current investigation, additional research directions should involve studying the safety and efficacy of these products across different stages of the disease and/or over longer periods of time. 

## 4. Materials and Methods

### 4.1. Animal Care

In the study, adults (12 weeks old, pre-symptomatic stage) male double transgenic APP/PS1 (B6.CBA-Tg) mice along with their wild-type (WT) littermates (Taconic Biosciences, Germantown, NY, USA and University of Oviedo, Oviedo, Spain) were used. The animals were housed in individually ventilated cages (IVCs) within the animal facility at the Advanced Research and Development Center for Experimental Medicine, “Prof. Ostin C. Mungiu”—CEMEX. They were maintained under standard husbandry conditions, including controlled room temperature (20 ± 4 °C), relative humidity (50 ± 5%), and a controlled light-dark cycle with unrestricted access to water and standard laboratory chow.

The experimental protocol and procedures were in compliance with the European Community Guidelines (Directive 2010/63/EU) and Romanian law (Law no. 43/2014) on the protection of animals used for scientific purposes. These measures underwent rigorous review and approval by both the Ethical Committee at “Grigore T. Popa” University of Medicine and Pharmacy of Iasi (no. 187/17.05.2022) and the National Sanitary Veterinary and Food Authority (no. 57/17.06.2022).

### 4.2. Reagents and Pharmacological Treatment

The selective CB2 receptor agonist JWH-133 was purchased from Tocris Bioscience^®^ (Bristol, UK). JWH133 was dissolved in a mixture of reagents (ethanol, polysorbate 80, and saline, 1:1:18) for an oral suspension. Donepezil, achieved as “Aricept” 10 mg orodispersible tablets from Pfizer, was ground into a powder form. An adequate quantity was then suspended in a 0.5% carboxymethyl cellulose sodium (CMC-Na) salt solution. The EU-GMP-certified *Cannabis sativa* L. with 15.6% THC and <1% CBD (dried inflorescence; a batch of Cannabixir^®^ Medium Flos, PZN: 7001905; Cansativa GmbH, Mörfelden-Walldorf, Germany) was finely ground using an electrical mortar grinder RM 200 (Retsch GmbH, Haan, Germany). The resulting powder was sifted using a 125-micron strainer (BSS Mesh No. 120), and an ample quantity of the compound was then dispersed in a 0.5% CMC-Na solution.

Polysorbate 80 was procured from Sigma-Aldrich in Saint Louis, USA, while the 0.9% saline solution was sourced from B. Braun Pharmaceuticals S.A., Timisoara, Romania. Ethanol was obtained from Chimreactiv SRL, Bucharest, Romania.

In our study, the selected doses were chosen to ensure a balance between safety, efficacy, and the need for further exploration of long-term effects. Specifically, for JWH-133, we opted for a dosing range aligned with findings from short-term studies in the literature [23,24,60]. As for Cannabixir^®^ Medium Flos, our dosing decision was based on both the LD50 data and the favorable pharmacokinetic profile identified in our previous research [22]. JWH-133 (0.2 mg/kg), Cannabixir^®^ Medium Flos (2.5 mg/kg), donepezil (0.65 mg/kg), and a 0.1% aqueous suspension of CMC-Na used as a vehicle were administered via gavage at a volume of 0.5 mL/100 g of body weight.

The mice (*n* = 36) were divided randomly into 6 experimental groups (either control or chronic cannabinoids therapy, 6 mice/drug condition), including the control (ConG) group, the JWH-133 (JG) group, the JWH-133 + donepezil (JDG) group, the Cannabixir^®^ Medium Flos (PG) group, the Cannabixir^®^ Medium Flos + donepezil (PDG) group, and the vehicle (WT) group.

In order to reproduce a model of chronic intermittent exposure to cannabinoids [61,62], mice were treated for 5 consecutive days, followed by 2 days without therapy, over 90 days. This approach was developed to mitigate the “on-target” adverse effects frequently observed with prolonged cannabinoid exposure, which may arise from continuous administration over an extended period [63,64].

### 4.3. Behavioral Evaluation of Cognitive Performance

To evaluate the behavior, Novel Object Recognition (NOR) and Elevated Plus Maze (EPM) tests were used. Their activity was recorded using the video-tracking software Smart 3.0 Basic Pack/Smart 3.0 SUPER (Harvard Apparatus, Holliston, MA, USA). Behavioral assessments were performed during the final 3 days of the treatment period. The NOR test was conducted in an open-field black acrylic arena (50 × 50 × 50), as previously reported [24]. The animals were introduced into the arena with two identical objects that were placed within a 10 cm circle, spaced at a fixed distance apart. Mice were then removed from the environment for a predetermined period of time, during which one of the two previously used (familiar) objects was substituted by a novel object, different in texture, appearance, and shape. The mouse’s ability to discriminate between the familiar and novel object was quantified as a novelty preference index (NPI), estimated as (TB − TA)/(TB + TA), where TA represents the time spent exploring the familiar object and TB denotes the time spent by the mouse with the novel object. Immediately after the test session phase of the NOR, for the EPM evaluation, mice were placed in a maze structured as a plus sign, elevated 50 cm above the ground, and their behavior recorded for 5 min. The total time spent in open arms was used as an indicator of anxiety.

### 4.4. PET-MRI

#### 4.4.1. PET-MRI Acquisition and Reconstruction

All animals underwent scanning using a small animal nanoScan PET-MRI, 1 Tesla (Mediso, Hungary), employing the mouse whole body coil with a 35 mm diameter. 

Before the PET-MRI scanning, mice received an intravenous injection of ^18^F-fluorodeoxyglucose (^18^F-FDG) in a maximum volume of 250 μL, with activity levels ranging between 9 and 22 MBq. Dynamic measurement of FDG uptake entailed capturing images immediately after injection and continuing for 30 min. The animals were kept under anesthesia during both radiotracer administration and PET-MRI scanning with 2% isoflurane. 

The MRI imaging protocol included a T1 GRE sequence in the axial and coronal planes, of similar resolution, with the following acquisition parameters: TR/TE: 12/3.3 ms; no. of excitations: 4; flip angle: 15°; thick slice: 2 mm. PET acquisition time was 30 min, with coincidence mode 1–5 and count rate in normal mode. The reconstruction parameters were as follows: 3D whole body protocol, fine resolution, matrix size 133 × 133 × 315 mm^3^ (voxel = 0.3 mm^3^); energy window 400–600 keV; coincidence mode: 1–5; reconstruction method: Tera-Tomo 3D; Basic Detector Model; no. of iterations: 8; no. of subsets: 3. 

#### 4.4.2. PET-MRI Data Processing

Quantification involved manually delineating whole brain volumes of interest (VOIs) on MRI slices for each animal, while PET parameters (such as SUVmean, SUVmax, TLG, and others) were automatically calculated using Nucline software version 2.01.017.

VOI statistics were generated for the whole brain area, and the mean standardized uptake values (SUVmean) were calculated for each animal. SUVmean = tissue activity concentration average (kBq/mL) × body weight (g)/injected dose (kBq). The average values of SUVmean for each group were calculated to compare the effect of a certain treatment on AD.

### 4.5. Sample Collection, Histology, and Immunohistochemistry Analysis

Following the completion of the testing period, the mice were euthanized via neck dislocation under deep isoflurane anesthesia. Subsequently, their brain tissues were harvested and prepared for analysis using histology and immunohistochemistry (IHC) techniques. The IHC staining followed previously reported protocols [65,66] using antibodies detailed in Table 2. 

Mouse brain tissues were post-fixed in a 10% formalin solution, dehydrated in graded alcohols, and cleared in xylene. Following the fixation step, the tissues were processed using the ExcelsiorTM AS Tissue Processor (Epredia Holdings Ltd., Portsmouth, NH, USA) and embedded in a paraffin wax block. Serial brain sections were made at 30 μm thickness using a semi-automatic microtome CUT 5062 (SLEE Medical GmbH, Nieder-Olm, Germany) and subjected to standard hematoxylin-eosin (HE) staining. HE-stained slides were examined via light microscopy using an Aperio AT2 DX slide scanner (Leica 557 GmBh, Berlin, Germany) at a magnification of 400×. For Aβ plaque quantification and immunoreactivity analysis, images were taken from at least 3 sections containing the cortex, hypothalamus, and hippocampus. Each sample received a numerical score ranging from 0 to 3, which assessed the expression of amyloid plaque pathology as follows: 0 (absence of plaques), 1 (1 to 5 plaques/mm^2^), 2 (6 to 20 plaques/mm^2^), or 3 (more than 20 plaques/mm^2^).

### 4.6. Statistical Analysis

A one-way ANOVA and unpaired Student’s *t*-test were used to assess variances among multiple independent groups and to determine significant differences between groups. The results are presented as the mean ± the standard error of the mean (SEM) and analyzed using Prism 7.0 (GraphPad Software 8, Boston, MA, USA). Statistical significance is represented by * when the *p*-value is <0.05. The experimental groups were compared against ConG and WT mice.

## 5. Conclusions

In conclusion, our current findings suggest that pharmacological activation of the expanded ECS via the selective CB2 agonist JWH-133 or Cannabixir^®^ Medium Flos—15.6% THC and <1% CBD ameliorates the Alzheimer-like phenotype in APP/PS1 mice by mitigating neuroinflammation and accumulation of Aβ plaque deposits, reducing cerebral glucose metabolism, and increasing glial reactivity. These results support the notion that targeting the ECS using cannabinoid receptor ligands, which lack psychoactive side effects, is a promising target for the development of novel therapeutic approaches against AD. However, further investigations are required to comprehend the mechanisms responsible for the Aβ-induced upregulation of the expanded ECS. To gain a more comprehensive understanding of the therapeutic implications of targeting the expanded ECS in AD, it will be essential to conduct treatment studies using selective compounds, either alone or in combination.

## Figures and Tables

**Table 1:** Quantitative immunohistochemistry (IHC) analysis of the biomarkers.

Biomarkers	Experimental Groups					
	ConG	JG	JDG	PG	PDG	WT
GFAP H	++	++	++	++++	+++	++++
GFAP SC	++	++	++	+++	++	+++
S100 H	++	++	++	++++	+++	+++
S100 SC	++	++	++	+++	++	++++
M1 AChR H	+	+++	+++	+++	++	++++
M1 AchR SC	+	+++	++	++	+++	++++
mTOR H	+++	++	++	++	++	+++
mTOR SC	++++	+	+	++	++	++
CB2 H	++++	++	++	+++	+++	+
CB2 SC	+++	++	++	+++	++	+

Average IHC positive cells/10 fields of 10,000 μm^2^; 1–6 IHC + positive cells; 6–12 IHC ++ positive cells; 12–18 IHC +++ positive cells; 18–24 IHC positive cells ++++; SC: subcortical area; H: hippocampus area; ConG: control group; JG: JWH-133 group; JDG: JWH-133 + donepezil group; PG: Cannabixir^®^ Medium Flos group; PDG: Cannabixir^®^ Medium Flos + donepezil group; WT: vehicle group; mTOR: mammalian target of rapamycin; M1mAChR: muscarinic acetylcholine receptor M1; CB2: cannabinoid receptor type 2.

**Table 2:** Antibodies, including both primary and secondary types, along with the corresponding dilutions utilized in immunohistochemical analysis.

Primary Antibody	Dilution	Secondary Antibody	Dilution
Anti-beta amyloid antibody (ab201060), Abcam (Cambridge, UK)	1:1000	Goat anti-rabbit	1:1000
Anti-canabinoid receptor antibodies (ACR-002), Alomone Labs (Jerusalem, Israel)	1:100	Goat anti-rabbit	1:100
GFAP (SYSY cat. no. 173002) SYSY Antibodies (Göttingen, Germany)	1:1000	Goat anti-rabbit	1:1000
Beta S100 ca48942 sab antibody, Sigma-Aldrich (St. Louis, MO, USA)	1:100	Goat anti-mouse	1:100
Anti-mTOR (ab109268), Abcam (Cambridge, UK)	1:100	Goat anti-rabbit	1:500
CHRM1 polyclonal antibody (PA5-90876), Thermo Fisher Scientific (Waltham, MA, USA)	1:100	Goat anti-rabbit	1:1000

## Data Availability

The datasets used and/or analyzed during the current study are available from the corresponding author upon reasonable request.
